# Designed to Be Green, Economic, and Efficient: A Ketone‐Ester‐Alcohol‐Alkane Blend for Future Spark‐Ignition Engines

**DOI:** 10.1002/cssc.202101704

**Published:** 2021-11-12

**Authors:** Philipp Ackermann, Karsten E. Braun, Patrick Burkardt, Sebastian Heger, Andrea König, Philipp Morsch, Bastian Lehrheuer, Maximilian Surger, Simon Völker, Lars Mathias Blank, Miaomiao Du, Karl Alexander Heufer, Martina Roß‐Nickoll, Jörn Viell, Niklas von der Aßen, Alexander Mitsos, Stefan Pischinger, Manuel Dahmen

**Affiliations:** ^1^ Institute of Energy and Climate Research IEK-10 Energy Systems Engineering Forschungszentrum Jülich GmbH Jülich 52425 Germany; ^2^ Process Systems Engineering RWTH Aachen University Aachen 52074 Germany; ^3^ Institute for Environmental Research RWTH Aachen University Aachen 52074 Germany; ^4^ Chair of Thermodynamics of Mobile Energy Conversion Systems RWTH Aachen University Aachen 52074 Germany; ^5^ Chair of High Pressure Gas Dynamics RWTH Aachen University Aachen 52074 Germany; ^6^ Institute of Applied Microbiology RWTH Aachen University Aachen 52074 Germany; ^7^ Institute of Technical Thermodynamics RWTH Aachen University Aachen 52062 Germany; ^8^ JARA-ENERGY Aachen 52056 Germany

**Keywords:** green chemistry, life-cycle analysis, model-based fuel design, renewable fuels, spark-ignition engines

## Abstract

Model‐based fuel design can tailor fuels to advanced engine concepts while minimizing environmental impact and production costs. A rationally designed ketone‐ester‐alcohol‐alkane (KEAA) blend for high efficiency spark‐ignition engines was assessed in a multi‐disciplinary manner, from production cost to ignition characteristics, engine performance, ecotoxicity, microbial storage stability, and carbon footprint. The comparison included RON 95 E10, ethanol, and two previously designed fuels. KEAA showed high indicated efficiencies in a single‐cylinder research engine. Ignition delay time measurements confirmed KEAA's high auto‐ignition resistance. KEAA exhibits a moderate toxicity and is not prone to microbial infestation. A well‐to‐wheel analysis showed the potential to lower the carbon footprint by 95 percent compared to RON 95 E10. The findings motivate further investigations on KEAA and demonstrate advancements in model‐based fuel design.

## Graphical abstract

**Renewable fuels**: Model‐based fuel design can tailor alternative fuels to advanced internal combustion engine concepts while minimizing production cost and global warming impact. A multi‐disciplinary assessment of KEAA, a rationally designed renewable fuel for high efficiency spark‐ignition engines, is presented. KEAA features promising production cost estimates, high engine efficiency, low environmental impact, and a low carbon footprint.

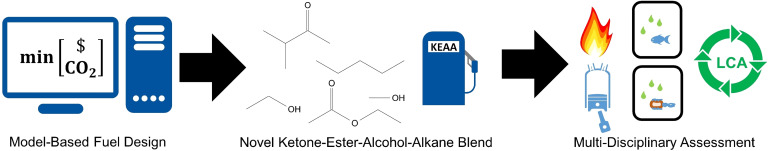

## Introduction

Three technologies have the potential to replace fossil fuels and enable climate‐neutral transportation: batteries, fuel cells, and renewable fuels. While batteries use renewable electricity in the most direct way and, like hydrogen fuel cells, produce no local CO_2_ emissions and pollutants, renewable fuels can readily be used in internal combustion engines and may allow re‐using today's fuel infrastructure for storage and handling. The most common fuels from renewable feedstocks in use today are bioethanol and biodiesel (a mixture of fatty acid methyl esters). As their current production from energy crops competes with food supply, they are not sustainable. Tremendous effort has thus been spent on researching the potential of lignocellulosic biomass and waste biomass as raw materials for biofuels.[^1^, ^2^, ^3^, ^4^] In addition, electrofuels that use carbon dioxide and hydrogen produced from renewable electricity have gained strong attention in both academia and industry.[^5^, ^6^] The cluster of excellence “The Fuel Science Center” (FSC) at RWTH Aachen University aims to develop fuels from all renewable resources, so‐called bio‐hybrid fuels. Furthermore, the FSC aims to tailor these bio‐hybrid fuels to advanced internal combustion engine concepts regarding efficient and clean combustion.

For advanced spark‐ignition (SI) engines, ethanol is an attractive fuel as it possesses a high knock‐resistance, which can be attributed to three properties: a high research octane number (RON), a high enthalpy of vaporization, and a high laminar burning velocity.^[7]^ It is often regarded as a prototype fuel for high‐efficiency SI engines.[^8^, ^9^] However, as a neat fuel, ethanol can cause problems in engine cold start and cold run as its high enthalpy of vaporization and low volatility complicate in‐cylinder mixture formation under challenging boundary conditions.[^8^, ^10^, ^11^]

**Figure 1:**
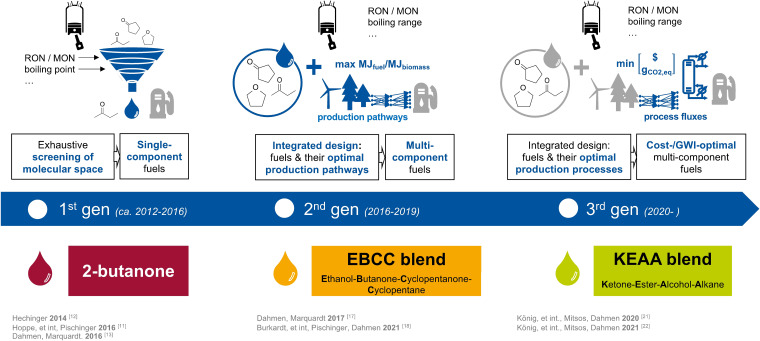
Evolution of model‐based fuel design as pursued within the Fuel Science Center at RWTH Aachen University: From single‐molecule fuel design (1st gen) over integrated design of fuel blends and their production pathways (2nd gen) to the integrated design of fuel blends and their production processes (3rd gen).

To find alternative SI fuels that overcome these limitations, we apply model‐based fuel design, using mathematical optimization to assemble a mixture of few, carefully chosen fuel components so that the mixture's properties are tailored to the needs of future engine concepts. The evolution of model‐based fuel design within the FSC is illustrated in Figure 1. Our earliest efforts focused on the rational design of single‐component fuels by computational screening of the molecular space and predicting key fuel properties like the boiling point or the auto‐ignition quality (Figure 1, left).[^12^, ^13^] This led to 2‐butanone, a promising fuel candidate for SI engines because of its high knock‐resistance and gasoline‐like enthalpy of vaporization.[^11^, ^12^] In single‐cylinder research engine experiments, 2‐butanone was found to provide strong efficiency increases and particle emission reductions compared to RON 95 E10 gasoline, similar in magnitude to ethanol.^[11]^ Moreover, oil dilution was found to be significantly lower compared to ethanol, which can be attributed to a better mixture formation under cold engine conditions.

Besides optimization of engine efficiency and emissions, renewable fuels aim at climate neutrality, and thus also their production must be optimized. Different rapid screening methods have been developed to identify promising fuel production pathways at an early development stage based on reaction stoichiometry, conversion, and selectivity data.[^14^, ^15^] Integrating mass‐based production pathway screening into the design of novel fuels allows to optimize the composition of multi‐component fuels with respect to aspects of both production and combustion simultaneously (Figure 1, center).[^16^, ^17^] One particularly promising blend identified by such an integrated product and production pathway design approach is EBCC, a mixture of ethanol, 2‐butanone, cyclopentanone, and cyclopentane,^[17]^ showing a similar combustion performance to neat ethanol and 2‐butanone in a single‐cylinder research engine with a compression ratio of 14.7.^[18]^

Whereas mass‐based production pathway screening can improve resource efficiency in fuel production, process network flux analysis (PNFA), an optimization‐based early process screening method, additionally allows to consider the energy demand of separation steps by employing thermodynamically sound short‐cut models.[^19^, ^20^] Recently, we have incorporated PNFA into our method for integrated design of renewable fuels to enable the design of production‐optimal fuels with tailored properties by directly minimizing the fuel production cost and global warming impact (GWI) (Figure 1, right).^[21]^ Furthermore, we have applied this new method to 47 pre‐screened fuel components as possible constituents of SI engine fuel and a corresponding large network of possible production pathways from renewable feedstocks.^[22]^ This analysis has resulted in a blend with a particularly promising trade‐off between fuel production costs and GWI: a ketone‐ester‐alcohol‐alkane (KEAA) blend composed of 40 mol % methyl isopropyl ketone, 25 mol % ethanol, 16 mol % methyl acetate, 13 mol % ethyl acetate, 4 mol % pentane, and 2 mol % methanol.^[22]^

In the present paper, we explore the potential of KEAA by leveraging a computational and experimental array of investigations to assess KEAA's properties, ranging from production cost and combustion behavior to ecotoxicity, microbial storage stability, and carbon footprint. In our assessment, we compare the performance of KEAA to those of our previously identified fuels 2‐butanone and EBCC as well as to those of ethanol, the prototype renewable SI engine fuel, and RON 95 E10. In this way, we illustrate the progress that the FSC fuel design efforts have been able to achieve over the past few years.

The remainder of this article is structured as follows: In the next section, for completeness, we briefly review the new PNFA‐based model‐based fuel design approach that led to the discovery of KEAA and then compare the production cost of the different fuels. Then, we assess the combustion performance, both in a rapid compression machine measuring fuel reactivity and in a single‐cylinder research engine measuring indicated efficiency and pollutant emissions. Following the combustion performance, we analyze the ecotoxicity and the microbial storage stability of the alternative fuels, before examining the carbon footprint based on a well‐to‐wheel life cycle assessment. Finally, we conclude with a discussion of the main findings and provide a brief outlook on further prospective work.

## Methods and Results

### Design of optimal renewable fuels: KEAA blend and its production performance

In our recent model‐based fuel design study,^[22]^ we investigated a list of 47 renewable fuel candidates as possible constituents for SI engine fuel. For that purpose, we assembled a large conversion pathway network connecting these species with renewable feedstocks based on an extensive literature review with the aid of the reaction databases reaxys and SciFinder.[^23^, ^24^] Lignocellulosic biomass, carbon dioxide from carbon capture, and hydrogen from renewable electricity were considered as feedstocks, and fossil gasoline was allowed as a blend component. Model‐based fuel design was stated as a mathematical optimization problem, where the mole fluxes through the pathway network and thus the resulting fuel composition are the degrees of freedom:^[22]^

$$\min\left\{\begin{array}{c} \mathrm{production}\;\mathrm{cost}\\ \mathrm{global}\;\mathrm{warning}\;\mathrm{impact} \end{array}\right\}s.t.\;\mathrm{PNFA}-\mathrm{based}\;\mathrm{production}\;\mathrm{pathway}\;\mathrm{model}\;\;\;\mathrm{product}\;\mathrm{and}\;\mathrm{by}-\mathrm{product}\;\mathrm{mole}\;\mathrm{balances}\;\;\;\mathrm{utility}\;\mathrm{requirements}\;\mathrm{of}\;\mathrm{reaction}\;\mathrm{and}\;\mathrm{separation}\;\mathrm{steps}\;\;\;\mathrm{costs}\;\left(\mathrm{raw}\;\mathrm{material},\;\mathrm{waste}\;\mathrm{disposal},\;\mathrm{utility},\;\mathrm{investment}\right)\;\;\;\mathrm{GWI}\;\mathrm{estimation}\;\mathrm{of}\;\mathrm{feedstock}\;\mathrm{and}\;\mathrm{process}\;\;\;\mathrm{fixed}\;\mathrm{amount}\;\mathrm{of}\;\mathrm{fuel}\;\mathrm{produced}\;\;\mathrm{fuel}\;\mathrm{property}\;\mathrm{model}\;\;\;\mathrm{mole}\;\mathrm{and}\;\mathrm{mass}\;\mathrm{fractions}\;\mathrm{of}\;\mathrm{fuel}\;\;\;\mathrm{fuel}\;\mathrm{property}\;\mathrm{models}\;\mathrm{and}\;\mathrm{mixing}\;\mathrm{rules}\;\;\;\mathrm{fuel}\;\mathrm{specification}$$

The fuel specification (i. e., upper and lower bounds for the physico‐chemical fuel properties) is shown in Table 1 and was chosen to facilitate high engine efficiency and clean combustion.^[22]^ In particular, the minimum required research octane number (RON) was set to 110 in the optimization problem, which is 15 points higher than the RON of a typical RON 95 gasoline fuel and about 8 points higher than that of a currently available premium pump fuel in Germany. To prevent problems in cold‐start and cold‐run operation such as excessive oil dilution, an upper bound for the enthalpy of evaporation of 60 kJ kg^−1^ air for a stoichiometric mixture was chosen. Additionally, to facilitate in‐cylinder mixture formation and thus cleaner combustion, limits on surface tension and viscosity were imposed. An oxygen content ≥10 wt % was selected to reduce soot formation propensity.

**Table 1:** Fuel specification used in the optimization problem together with properties of the alternative fuels KEEA, EBCC, 2‐butanone, and ethanol. Dash denotes no constraint; parentheses denote generic constraints given to the optimizer for numerical reasons. All property values have been calculated with the models described in refs. [21,22].

Property	Lower bound	Upper bound	KEAA^[a]^	EBCC^[b]^	2‐Butanone	Ethanol
RON	110	(150)	110	107	111	109
MON	–	–	102	92	106	90
enthalpy of combustion [MJ kg^−1^]	–	–	28.7	31.5	31.5	25.5
liquid density at 15 °C [kg m^−3^]	–	–	831	817	811	795
oxygen content [wt %]	10	(100)	27	23	22	35
vapor/bubble point pressure at 37.8 °C [kPa]	35	100	35	14	22	16
fuel fraction evaporated at 70 °C [mol %]	3	52	22	15	0	0
fuel fraction evaporated at 100 °C [mol %]	46	(100)	100	86	100	100
fuel fraction evaporated at 150 °C [mol %]	75	(100)	100	100	100	100
olefin content [vol%]	(0)	18	0	0	0	0
aromatic content [vol%]	(0)	35	0	0	0	0
kinematic viscosity at 25 °C [mm^2^ s^−1^]	0.5	2	0.75	1.02	0.49	1.36
enthalpy of vaporization at 25 °C [kJ kg_air_^−1^]	(0)	60	52	60	45	103
surface tension at 25 °C [mN m^−1^]	(0)	30	23	24	24	22

[a] Composition: 40 mol % methyl isopropyl ketone, 25 mol % ethanol, 16 mol % methyl acetate, 13 mol % ethyl acetate, 4 mol % pentane, 2 mol % methanol. [b] Composition: 50 mol % ethanol, 21 mol % 2‐butanone, 15 mol % cyclopentane, 14 mol % cyclopentanone.

**Figure 2:**
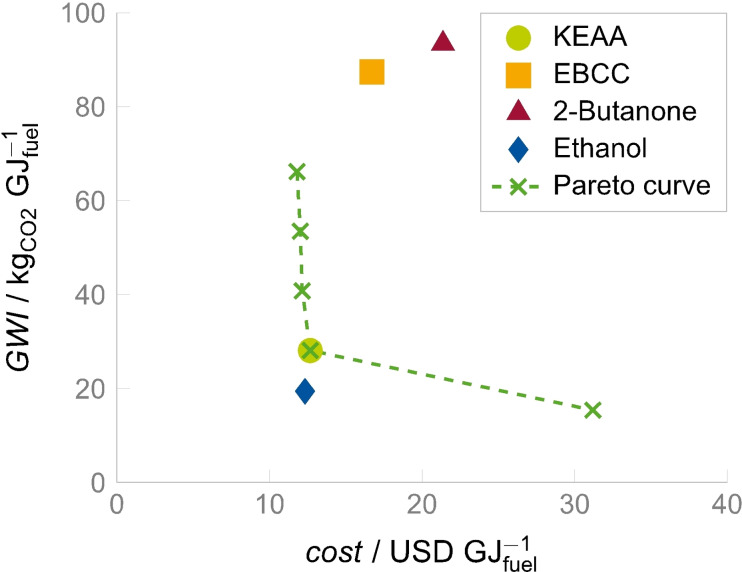
Pareto curve of optimal designs (reproduced from Ref. [22]): Each cross denotes an optimal design; the dashed lines are added to guide the eye. Cost and GWI data for ethanol and 2‐butanone from PFNA have been reproduced from Ref. [22]. Data for EBCC have been computed based on the PNFA model from Ref. [22].

The nonlinear optimization problem was implemented and solved in GAMS using the deterministic global solver BARON version 19.12.7.[^25^, ^26^] To investigate the trade‐off between the two objectives, cost minimization and GWI minimization, the epsilon‐constraint method was used.^[27]^ Figure 2 depicts the Pareto‐optimal solutions to the optimization problem. Each cross represents a different optimal fuel blend and its corresponding process configuration. The fuel blend with a particularly promising trade‐off between production costs and GWI is a fully renewable blend referred to as KEAA, a six‐component mixture rich in methyl isopropyl ketone (full composition given in Table 1).

**Scheme 1:**
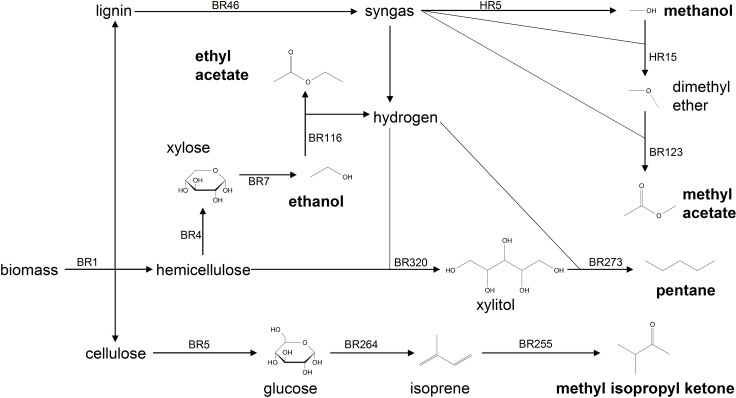
Overview on the production pathways that yield KEAA (reproduced from Ref. [22]).

As can be seen from Scheme 1, the only feedstock selected by the optimizer to produce KEAA is lignocellulosic biomass, where the cellulose fraction is converted to methyl isopropyl ketone, the hemicellulose fraction is converted to ethanol, ethyl acetate, and pentane, and the lignin fraction is gasified to syngas and converted to methanol and methyl acetate. Note that hydrogen is needed for upgrading of intermediates but is provided internally in both the reaction step from ethanol to ethyl acetate and the gasification of lignin.

For comparison, the production performance of ethanol and our two previously designed fuels 2‐butanone and EBCC has been evaluated with PNFA using the same reaction network and the same economic and ecological parameters applied in Ref. [22] (cf. Figure 2).^[22]^ Notably, KEAA is close to ethanol in terms of production cost and GWI, whereas EBCC and 2‐butanone are significantly behind. These results illustrate that by integrating PNFA into model‐based fuel design we have improved considerably in terms of production performance (from 2‐butanone to EBCC and to KEAA), but at the same time the results highlight that neat ethanol is very difficult to beat cost‐wise. Note, however, that due to its high enthalpy of vaporization and low volatility neat ethanol does not constitute a feasible solution to the optimization problem (see Table 1).

### Combustion performance

#### Auto‐ignition in a rapid compression machine

To assess the reactivity of KEAA, we performed ignition delay time (IDT) measurements in a rapid compression machine (RCM). The RCM data allows to compare the ignition propensity of the different fuels while disregarding engine specific effects like fuel vaporization or in‐cylinder flow and mixing. For a description of the experimental setup, see the Supporting Information about experimental and computational methods.

**Figure 3:**
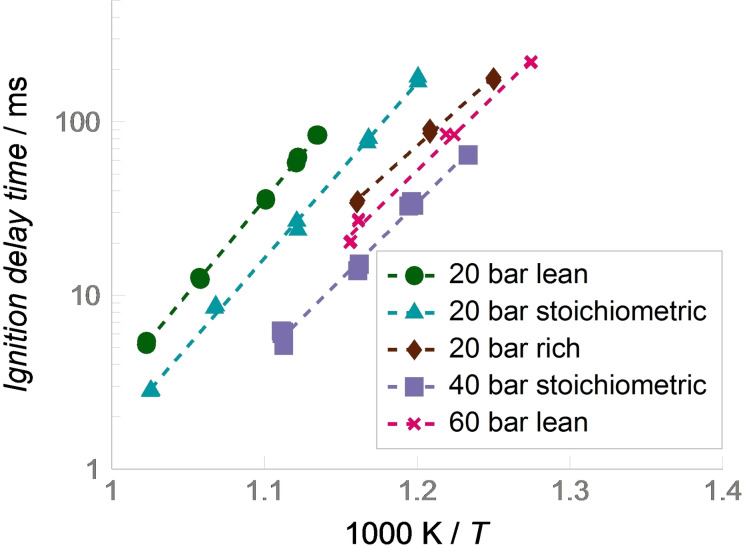
Ignition delay times of KEAA measured in a RCM. Dashed lines have been added to guide the eye of the reader. The slope of the line corresponds to the global activation energy.

As future high‐efficiency engines are expected to operate under highly boosted and lean conditions, we first assessed the dependence of the IDT on pressure and air/fuel equivalence ratio. For that purpose, five different data sets were measured, one for each of the experimental conditions given in Table 2. The investigated temperature range was limited either by the maximum reachable temperature with 100 % argon as diluent or by the minimum measurable IDT of 2 ms. The determined IDTs for all five data sets are presented in Figure 3. Note that IDTs are shown in a typical log‐linear Arrhenius‐type plot where the linear *x*‐axis shows thousand times the inverse of the temperature after compression and the *y*‐axis shows the IDT on a logarithmic scale. The experimental scatter of 20 % is presented as error bars. For all investigated data sets, the IDT decreases exponentially with increasing temperature, leading to a straight line in the Arrhenius‐type plot.

**Table 2:** Composition of KEAA mixtures investigated in the RCM in a temperature regime of 780 to 980 K. Fuel/air equivalence ratios below 1 correspond to lean mixtures, whereas those greater than 1 correspond to rich mixtures.

End of compression pressure [bar]	Fuel/air equivalence ratio	Nitrogen/diluent	Argon/diluent
20	0.5	0.0	1.0
20	1.0	0.0	1.0
20	2.0	0.0	1.0
40	1.0	0.0	1.0
60	0.5	1.0	0.0

Comparing the 20 bar data sets with varying fuel/air equivalence ratio, it can be noted that the reactivity increases with increasing equivalence ratio. The lean data set shows the longest IDTs and the rich one shows the shortest. Interestingly, the global activation energy for the rich conditions differs from those of the stoichiometric and lean data sets, thus indicating a change in dominating reaction pathways.

The influence of pressure on the IDTs was captured for stoichiometric and lean conditions, where the pressure after end of compression was increased to 40 and 60 bar, respectively. As expected, IDTs decrease with higher pressure. Overall, the lean mixture at 60 bar is slightly less reactive than the stoichiometric mixture at 40 bar. This can be explained by the opposing effects of pressure increase and decrease of equivalence ratio. For both data sets with higher pressure, the global activation energy decreases compared to the 20 bar data set, indicating a change in dominating reaction pathways. This effect is more pronounced for the 60 bar data set, indicating a continuous pressure dependency of the global activation energy.

**Figure 4:**
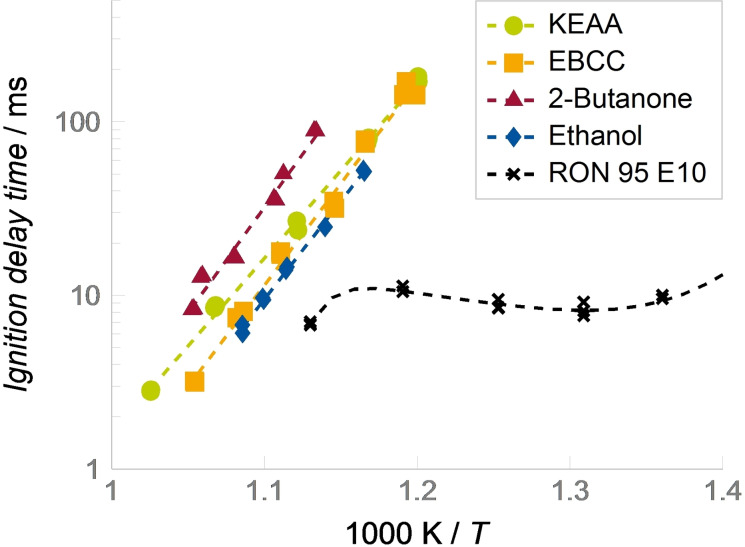
Comparison of ignition delay times between the different fuels. Experiments were measured at 20 bar and stoichiometric conditions. Dashed lines represent fits through the data points.

A comparison of the stoichiometric 20 bar data set to previously collected data for other fuels and fuel blends at similar conditions is presented in Figure 4. It gives meaningful insights into the qualitative ignition behavior of the investigated fuels and thus allows for a reactivity‐based ranking. However, the findings cannot be directly translated to a full‐size engine, where the ignition is a superposition of chemical kinetics and physical effects.

In our comparison, the market‐grade RON 95 E10 takes a special role as it is the only fuel that shows a negative temperature coefficient (NTC) behavior, that is, between 750 and 850 K the ignition delay times actually increase with increasing temperature (see the S‐shaped curve in Figure 4). The NTC behavior indicates the transition from typical high‐temperature fuel decomposition to low‐temperature pathways. As no NTC regime is observed for the other fuels, they do not exhibit significant low‐temperature chemistry in the investigated regime.

Neat 2‐butanone shows the longest IDTs, meaning it has the lowest reactivity. Neat ethanol, EBCC, and KEAA show similar global activation energies; however, EBCC and KEAA exhibit slightly longer IDTs than ethanol and are thus considered less reactive. While EBCC and KEAA have a similar reactivity at approximately 830 K, KEAA is less reactive for higher temperatures, where it approaches the reactivity of 2‐butanone. However, as the global activation energy of KEAA is lower than that of 2‐butanone, IDT reductions with increasing temperature are less strong compared to 2‐butanone. A quantitative comparison to market‐grade RON 95 E10 is hardly feasible as the ignition is dominated by the NTC behavior in this temperature regime. Nevertheless, RON 95 E10 is clearly the most reactive of the considered fuels indicating a comparatively low knock resistance. In summary, the RCM results indicate a high knock‐resistance for KEAA as it has a generally low reactivity that also does not increase swiftly with higher temperature.

#### Combustion behavior in a single‐cylinder engine

**Figure 5:**
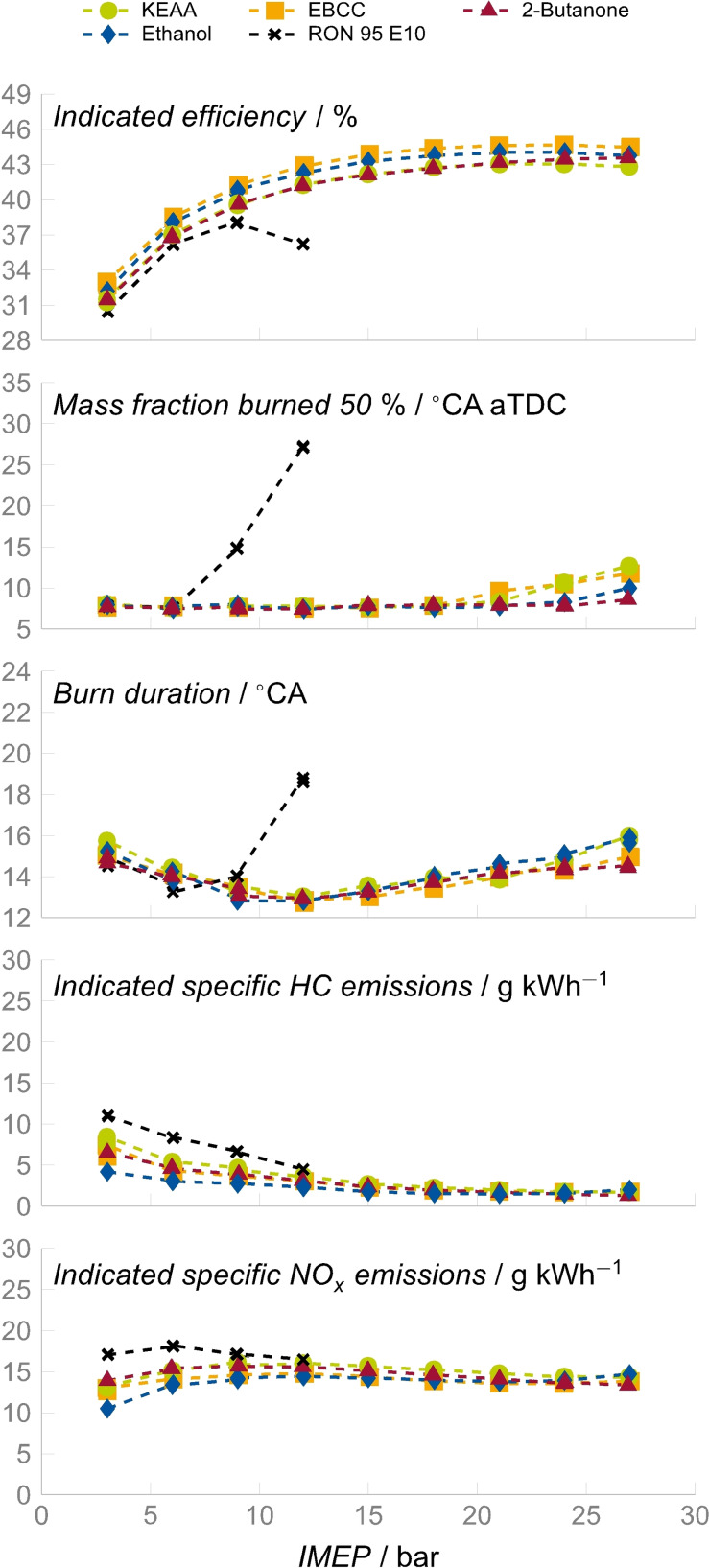
Results of the load variation: plotted are over the IMEP: the indicated efficiency, MFB50 (the crank angle after top dead center at which 50 % of the mass fraction is burned), the burn duration, and the indicated specific hydrocarbon and NO_*x*_ emissions. The engine was operated at an engine speed of 2000 min^−1^ and air/fuel ratio of 1.0; with start of injection at 300° crank angle before top dead center, intake temperature of 25 °C, and coolant temperature of 90 °C.

We performed experiments on a single‐cylinder research engine with a compression ratio (CR) of 16.4 to evaluate the engine performance of KEAA regarding the indicated efficiency and the pollutant emissions during load variation. The experimental setup is described in detail in the Supporting Information about experimental and computational methods. The results are visualized in Figure 5. The engine load is quantified by the indicated mean effective pressure (IMEP), which is the indicated work supplied by the engine divided by the displacement volume. When comparing the single fuels with respect to their indicated efficiency (*η*_i_), it can be noted that EBCC achieves the highest *η*_i_ followed by neat ethanol. The indicated efficiencies of both KEAA and neat 2‐butanone are almost indistinguishable and slightly lower than those of EBCC and ethanol. In contrast, the indicated efficiency of RON 95 E10 is the lowest among all the investigated fuels. Moreover, the maximum achieved IMEP for RON 95 E10 is only 12 bar and thus 15 bar lower than for the alternative fuels. This restriction is a direct consequence of the low knock resistance of RON 95 E10. Although the knock resistance of a fuel is linked to its reactivity, the order of the indicated efficiencies in Figure 5 does not match with the order of reactivities in Figure 4. Thus, there are other effects that influence the combustion process.

To further assess the trends in the indicated efficiency, the combustion parameters mass fraction burned (MFB) and burn duration are also shown in Figure 5. For 50 % of the mass fraction burned (MFB50), a value between 7 and 8° crank angle (CA) after top dead center (aTDC) was targeted. RON 95 E10 can maintain this target value only up to IMEP=6 bar. At IMEP=9 bar and above, MFB50 must be retarded to avoid knocking combustion. This attenuates the *η*_i_ differences compared to the alternative fuels, which are able to maintain the target level for MFB50 up until IMEP=18 bar. At IMEP=21 bar and higher, EBCC and KEAA show a more retarded MFB50 than the neat fuels ethanol and 2‐butanone. Thus, the blends show a more pronounced knock restriction than the neat alternative fuels.

The burn duration, which describes the time between MFB10 and MFB90, depends on both the laminar burning velocity and the location of MFB50. In case of RON 95 E10, the burn duration increased when MFB50 is retarded. An increase in both MFB50 and burn duration lowers the indicated efficiency. Thus, the decrease of the indicated efficiency of RON 95 E10 at IMEP=12 bar can be explained. The burn durations of the four other fuels show only slight differences as long as MFB50 is equal. At IMEP=27 bar, where MFB50 shows the largest differences between the alternative fuels, both neat 2‐butanone and EBCC have shorter burn durations than neat ethanol and KEAA. In case of 2‐butanone, this behavior can be attributed to the advanced MFB50. For EBCC, a presumably higher laminar burning velocity could explain the relatively short burn duration.

When considering both MFB50 and burn duration, only in case of 2‐butanone an influence on the indicated efficiency can be observed. Since 2‐butanone shows both the most advanced MFB50 and the shortest burn duration, its indicated efficiency increased between IMEP=21 and 27 bar compared to those of the other fuels. However, 2‐butanone cannot achieve the highest indicated efficiency overall.

An explanation for the lowest indicated efficiency in case of RON 95 E10 even at low IMEP can be found in the indicated specific hydrocarbon (ISHC) emissions shown in Figure 5, where RON 95 E10 shows higher ISHC emissions than all the alternative fuels. This behavior can be attributed to the low oxygen content of RON 95 E10, which makes hydrocarbon oxidation less effective and thus accounts for the higher amount of unburned hydrocarbons. The importance of the oxygen fraction for the hydrocarbon oxidation can also be observed for the alternative fuels. Ethanol has the highest oxygen content of all the investigated fuels and correspondingly shows the lowest ISHC emissions. Note that the ISHC emissions of the different fuels decrease with higher IMEP, as the temperatures of both combustion and exhaust gas increase, which reduces the flame quenching and improves the post oxidation.

The indicated specific nitrogen oxides (ISNO_*x*_) emissions depend on both the combustion temperature and the time the critical temperature of 2000 K is exceeded. Here, lower combustion temperatures lead to lower ISNO_*x*_ emissions. Up to IMEP=12 bar, ethanol shows the lowest ISNO_*x*_ in Figure 5. This is caused by both ethanol's high enthalpy of vaporization and its low adiabatic flame temperature (2193 K). RON 95 E10 shows the highest ISNO_*x*_ emissions, since its enthalpy of vaporization is low and its adiabatic flame temperature is high (2275 K). Even the retarded MFB50 of RON 95 E10 cannot compensate for the impact of these properties. At IMEP>12 bar, ethanol does not show the lowest ISNO_*x*_ emissions anymore. In this specific IMEP range, the engine operates with an externally boosted intake pressure to achieve the desired IMEP. Since the boost pressure depends on both the combustion efficiency and the fuel's oxygen content, it can individually influence the charge motion and thus the occurrence of locally produced ISNO_*x*_ emissions for each fuel.

Further assumptions of the combustion process can be derived from the fuel properties. The high enthalpy of vaporization of ethanol results in decreased wall heat losses and consequently in an increased efficiency. Moreover, since cyclopentane has no oxygen content, EBCC has a high stoichiometric air requirement. A high stoichiometric air requirement results in low pumping losses, since more fresh air is required to burn the fuel. A dedicated combination of both effects could contribute to the highest indicated efficiency in case of EBCC. Although EBCC was the best‐performing fuel in the engine investigations, KEAA also performed well and is considered a promising fuel for engine application.

### Ecotoxicity and microbiological stability

#### Ecotoxicity

The toxicity of the renewable fuels on aquatic organisms was assessed to determine the half maximal concentration (EC50), a fuel blend concentration at which 50 % of the adverse effect occur. High EC50 values indicate a low toxicity and an ecotoxicologically promising candidate. As an initial assay the acute immobilization test was performed using *Daphnia magna* STRAUS (clone 5).^[28]^ For a comprehensive description of the test procedure, see the Supporting Information about experimental and computational methods. While a complete toxicity assessment of alternative fuels consists of a more diverse test battery, involving several trophic levels,^[29]^ the chosen bioassay provides a first and quick insight regarding toxicity potential.

**Figure 6:**
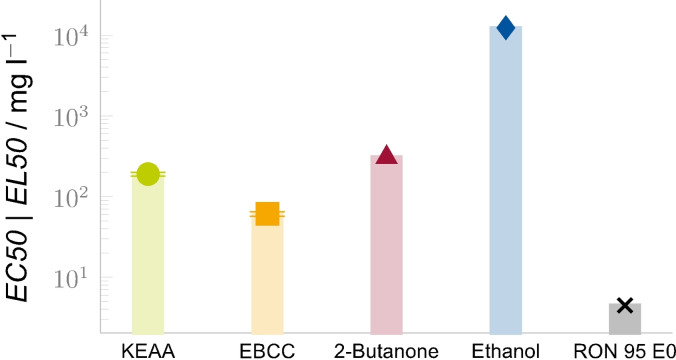
Ecotoxicological assessment of the fuels in the acute *Daphnia* immobilization assay: for RON 95 E0 gasoline the 50 % effect load level was determined, for the alternative fuels the 50 % effect concentrations. For KEAA and EBCC the 95 % confidence level of the experimental data for is shown, for the remaining fuels literature data is shown. Higher values indicate lower toxicity.

Gasoline has a complex composition and low solubility in water; thus, its toxicity is usually not tested directly but instead by creating a water‐accommodated fraction. The water phase, containing mixable and soluble gasoline fractions, is then used in the assessment. This procedure yields an effect load level (EL), which replaces the effect concentration as a classification criterion.^[30]^ After 48 h, pure gasoline has an EL50 of 4.5 mg L^−1^ in the acute *Daphnia* immobilization test,^[31]^ while the analyzed alternative fuels showed EC50 values of 61.0 mg L^−1^ (EBCC), 189.6 mg L^−1^ (KEAA), 308 mg L^−1^ (2‐butanone), and 12 340 mg L^−1^ (ethanol), as shown in Figure 6.

The EC50 of KEAA is two orders of magnitude lower than the EC50 of ethanol and the EL50 of gasoline is again two orders of magnitude lower than the EC50 of KEAA. These results indicate that KEAA has a significantly higher toxic potency on aquatic organisms than neat ethanol (or neat 2‐butanone); however, its toxic potency is significantly lower than the toxic potency of gasoline. In comparison to EBCC, KEAA is about three times less toxic in the performed assay.

Purely based on the generated acute single‐species toxicity data, EBCC would be classified under the globally harmonized system of classification and labelling of chemicals as H412, “harmful to aquatic life with long‐lasting effects”. KEAA, in contrast, would fall under the more lenient H413, “may cause long‐lasting harmful effects to aquatic life”, the latter being the lowest hazard level for environmental hazards besides non‐hazardous.^[32]^

#### Microbiological stability

**Figure 7:**
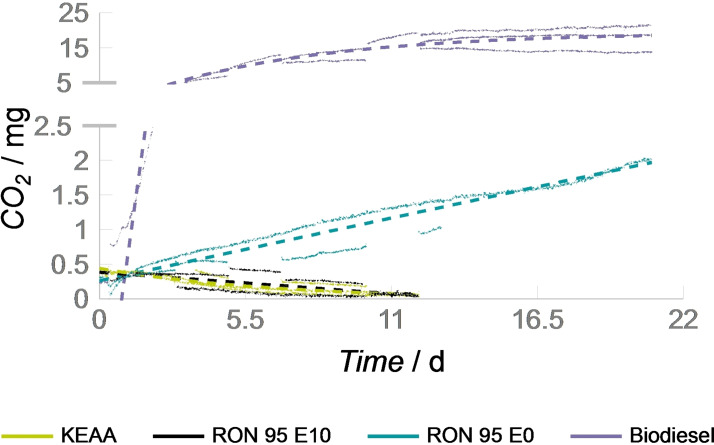
Microbial activity on KEAA, gasolines, and biodiesel. Shown is the accumulation of microbially produced CO_2_. Continuously and discontinuously measured values of three independent biological replicates per fuel are shown as points. The trend per fuel is highlighted as a dashed line.

The microbial stability of KEAA is measured and compared to two reference gasolines RON 95 E0 and RON 95 E10 with different ethanol content (0 and 10 vol %, respectively), and biodiesel (rapeseed oil methyl ester, RME). Contrary to the other sections we compare the performance of KEAA to biodiesel and a former ethanol‐free commercial gasoline, RON 95 E0. Biodiesel is an alternative fuel that is prone to microbial contamination and thus used as an active control. RON 95 E0 is an SI fuel where microbial activity can also be observed. To evaluate the storage stability, the fuels were exposed to a defined mixture of microbes within a free water phase representative of contaminated fuel storage.^[33]^ Microbially produced CO_2_, the endpoint of microbial metabolic activity, was measured as indication of microbial activity (Figure 7). A detailed description about the strains and the experimental setup can be found in the Supporting Information.

Biological replicates exposed to RON 95 E0 yield 2 mg CO_2_ in 300 mL headspace within three weeks. In contrast, the positive control biodiesel (RME) yields 20 mg CO_2_ in 300 mL headspace. Thus, RON 95 E0 showed one‐tenth the microbial activity possible in the positive control biodiesel (RME) and has a correspondingly higher microbial storage stability. This can be attributed to the better nutrient supply of the biodiesel. The fatty acid methyl esters represent simpler carbon sources due to the compound class and their chain length (C_18_). In contrast, RON 95 E0 consists of C_4_−C_7_ alkanes, alkenes, ethers, and aromatics, which are much more difficult for microbes to access as carbon sources. Furthermore, due to its plant origin, biodiesel has the potential for contamination with additional nitrogen and phosphorus sources.

No increase of CO_2_ in the headspace was detected for the currently commercially available gasoline RON 95 E10 and the here reported fuel KEAA. RON95 E10 and KEAA have high alcohol contents, which is a likely cause for the strongly reduced metabolic activity. High‐performance liquid chromatography refractive index (HPLC‐RI) analysis confirmed very high alcohol concentrations in the free water phase. In the case of E10 an ethanol concentration of 29.4 vol % (5 m) and in the case of KEAA an ethanol concentration of 17.7 vol % (3 m) as well as a methanol concentration of 4.1 vol % (1 m) are present in the free water phase, respectively (Table 3). This eliminates the already low microbial susceptibility of gasoline fuels completely, and no signs of microbial activity could be recorded over the course of two weeks. However, for RON 95 E10 and KEAA, instead of a constant CO_2_ value of 0.2–0.3 mg per 300 mL headspace (0.04 vol %, ambient air), a continuously decreasing CO_2_ concentration was measured. This decrease can be attributed to the dilution of the headspace with alcohols and thus lead to a reduced CO_2_ value.

**Table 3:** HPLC analysis of water phases below fuels during assessment of microbial storability. Listed are the established concentrations of ethanol, methanol, and acetate methyl ester in control samples after one week. The concentrations are mean values of two replicates.

Fuel	Concentration [M]		
	Ethanol	Methanol	Methyl acetate
RON 95 E0	0	0	0
RON 95 E10	5.095	0	0
KEAA	3.200	1.004	0.8477
biodiesel	0	0.035	0

### Life cycle assessment

We performed a prospective well‐to‐wheel life cycle assessment (LCA) with focus on the carbon footprint to compare KEAA to 2‐butanone, EBCC, ethanol, and RON 95 E10 pump fuel. For this purpose, we analyzed the carbon footprint for the “provision of 1 MJ of enthalpy of combustion” as the functional unit in a best‐case and worst‐case scenario. The best‐case scenario considers electricity‐based technologies for heat supply and electricity from wind power, whereas the worst‐case scenario uses today's conventional technologies for heat supply and electricity from the German power grid. For more details on the LCA methodology, the scenarios, and the considered process data, we refer to the Supporting Information.

**Figure 8:**
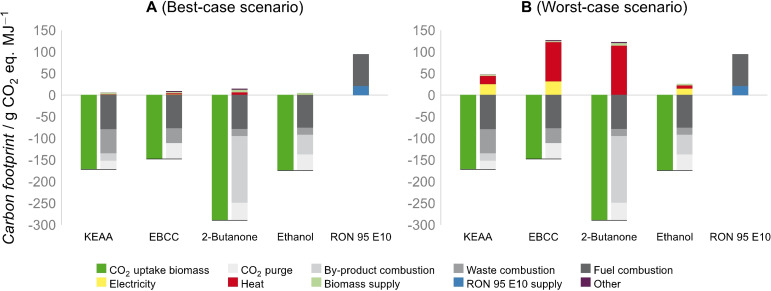
Well‐to‐wheel carbon footprints of KEAA, EBCC, 2‐butanone, and ethanol compared to the one of RON 95 E10 in g CO_2_ eq. MJ^−1^ for the (A) best‐case and (B) worst‐case scenario. The carbon footprint of the carbon uptake due to biomass utilization is negative (dark green). Positive carbon footprints result from CO_2_ emissions in purge gases from fuel production (light grey), the combustion of the by‐products lignin and hemicellulose as well as of wastes (medium grey), fuel combustion (dark grey), electricity (yellow), heat (red), biomass (light green), RON 95 E10 supply (blue), and minor emissions due to wastewater treatment and process and cooling water supply (violet).

In the best‐case scenario (Figure 8A), the carbon footprints of all the alternative fuels are way below the carbon footprint of fossil gasoline RON 95 E10, which is 94 g CO_2_ eq. MJ^−1^. Ethanol has the lowest carbon footprint with 4 g CO_2_ eq. MJ^−1^, followed by KEAA with 5, EBCC with 8, and 2‐butanone with 14 g CO_2_ eq. MJ^−1^, respectively. Electricity and heat supply contribute only slightly to the overall carbon footprint since their carbon footprints are very low in the best‐case scenario.

In the worst‐case scenario (Figure 8B), differences between the four fuels are greater than in the best‐case scenario, since electricity and heat are supplied via today's conventional technologies (yellow and red, Figure 8). The carbon footprints of both ethanol (26 g CO_2_ eq. MJ^−1^) and KEAA (47 g CO_2_ eq. MJ^−1^) are still below the one of fossil gasoline. In contrast, 2‐butanone (122 g CO_2_ eq. MJ^−1^) and EBCC (126 g CO_2_ eq. MJ^−1^) perform worse than fossil gasoline since both fuels require large amounts of heat with 1.1 and 0.9 MJ per MJ of enthalpy of combustion, respectively.

The results in Figure 8 show that KEAA and ethanol are promising fuels in both scenarios, while 2‐butanone and EBCC are only suitable in the best‐case scenario. Irrespective of the considered scenario, EBCC uses biomass most efficiently with 85.3 g of biomass per MJ of enthalpy of combustion, resulting in the lowest carbon footprints due to the carbon uptake and the supply of biomass (dark and light green, Figure 8). In contrast, 2‐butanone uses biomass least efficiently with 167.0 g of biomass per MJ of enthalpy of combustion. Consequently, 52 % of the carbon is transformed into the fuel in case of EBCC, whereas only 27 % of the carbon ends up in 2‐butanone (dark grey, Figure 8). For KEAA and ethanol, 46 and 43 % of the carbon is transformed into the fuel, respectively. In the use‐phase (i. e., fuel combustion), all the alternative fuels emit about 75–78 g CO_2_ eq. MJ^−1^, which is slightly more than the 74 g CO_2_ eq. MJ^−1^ of fossil gasoline (dark grey, Figure 8).

Note, however, that apart from waste combustion and wastewater treatment, no further waste treatment processes are considered in this study. This assumption is made because for most reaction pathways only yields are published with no information on by‐products. Moreover, we consider the end‐of‐life of the by‐products lignin and hemicellulose but neglect that both could serve as valuable substances in other industrial processes. Especially 2‐butanone production leads to large amounts of these by‐products. Therefore, alternative uses for lignin and hemicellulose should be investigated in proceeding analyses.

## Discussion

A renewable KEAA blend for SI engines was identified by means of model‐based fuel design. Through an integrated product and process design, fuel production cost and global warming impact were minimized while ensuring compliance with a fuel specification that aims at efficient and clean combustion in a future SI engine.

Based on PNFA, KEAA's fuel production costs were estimated to be in the ballpark of neat ethanol and significantly lower than those of our two previously designed fuels 2‐butanone and EBCC. In RCM investigations, KEAA, like 2‐butanone, ethanol, and EBCC, was found to exhibit distinctively longer chemical ignition delays than RON 95 E10 without any indication of low‐temperature combustion chemistry. In line with this, engine testing showed very high knock‐resistance and thus efficiency gains compared to RON 95 E10 for all the alternative fuels. More specifically, KEAA achieved a maximum indicated efficiency of 43.1 %, 5 % higher than that of RON 95 E10 and in the range of that of 2‐butanone. Maximum indicated efficiencies for EBCC and ethanol were even higher, with gains of 1.5 and 0.9 % compared to KEAA, respectively. The higher efficiency of ethanol can be attributed to its high laminar burning velocity (LBV) and its higher enthalpy of vaporization (109 kJ kg_air_^−1^).^[34]^ The latter results in charge cooling and thus provides a higher knock resistance.^[8]^ Similarly, the higher efficiency of EBCC can be attributed to a slightly higher enthalpy of vaporization and the high LBV of cyclopentanone.^[35]^ The LBV of cyclopentanone is even higher than that of ethanol, which can explain the efficiency gains of EBCC relative to ethanol. In contrast, KEAA contains significant shares of methyl isopropyl ketone and two esters with moderate LBVs.^[36]^ We note, however, that no dedicated engine performance optimization for KEAA was performed as this study aimed at a comparison of the different fuels under identical conditions.

The environmental performance of KEAA is much better than that of the previously identified EBCC blend. More specifically, an LCA showed that KEAA, but not EBCC and 2‐butanone, has a lower carbon footprint than RON 95 E10 in a worst‐case scenario based on today's conventional technologies for heat supply and the current electricity mix. KEAA is only surpassed by ethanol, whose carbon footprint is roughly 50 % smaller. Note that these results are qualitatively in good agreement with the GWI values from PNFA, the method behind the mathematical optimization that yielded KEAA. In the best‐case scenario with electricity‐based technologies for heat supply and wind power electricity, all the alternative fuels show low carbon footprints. However, again, KEAA outperforms EBCC and 2‐butanone and is approximately on par with ethanol.

While the carbon footprint is a key aspect of the environmental performance of a fuel, accidental environmental fuel release can cause direct harm to both humans and the ecosystem. We therefore also investigated the ecotoxicity of the alternative fuels. In the acute *Daphnia* immobilization assay, KEAA revealed a moderate toxic potency that is far below that of RON 95 E0 and also below that of EBCC. With its high alcohol content KEAA also has a high microbial storage stability and thus is not prone to microbial infestation, a known problem with biodiesel.

While the investigations showed that KEAA is a promising fuel candidate for high‐efficiency SI engines, ethanol is still slightly ahead in the comparative analysis. Ethanol, however, does not meet the vapor pressure, fuel fraction (mol %) evaporated at 70 °C, and enthalpy of vaporization requirements used in our design study (Table 1). These property constraints have been imposed to facilitate in‐cylinder mixture formation under challenging boundary conditions, particularly during engine cold run.[^10^, ^11^] Further investigations of the fuels under conditions representative of cold engine operation need to be conducted to clarify whether KEAA indeed outperforms ethanol in this regard.

## Conclusion

We conclude that ketone‐ester‐alcohol‐alkane (KEAA) needs to be studied in more detail and in more diverse ways to determine its potential more accurately. From a combustion point of view, further fundamental research on KEAA's properties and engine optimization are required to clarify the full potential of this fuel. Research should also be directed towards the potential of blending KEAA with fossil gasoline, as blending could help to overcome the significant market entry barriers inherent to the introduction of 100 %‐renewable fuel for dedicated engines. Along the same lines, further studies on fuel handling are necessary. While KEAA showed to be stable against microbial infestation, future research should also address material compatibility. At the same time, the production pathways involved need to be examined more closely so that an initial conceptual process design and optimization can be carried out as a basis for a techno‐economic assessment and more detailed life cycle assessment (LCA). The ecotoxicity also needs to be examined further using a more complete test battery.^[29]^

The cost reductions and the improved carbon footprints from 2‐butanone, to ethanol–2‐butanone‐cyclopentanone‐cyclopentane (EBCC), and finally KEAA clearly illustrate the progress achieved through successively expanding our integrated design methodology (Figure 1). The multi‐disciplinary assessment also points the way to further improvements in the model‐based fuel design approach. Adding the laminar burning velocity (LBV) to the optimization problem would provide another lever to design fuels for extreme combustion efficiency. A promising development in this respect is a recently published artificial neural network‐based prediction model for LBV from molecular structure.^[36]^ Simultaneous optimization of a fuel's auto‐ignition delay, LBV, and enthalpy of vaporization might enable outperforming ethanol even more strongly than EBCC could do in this study. Our model‐based fuel design method already allows to design blends of renewable fuel and fossil gasoline that fulfill key aspects of today's gasoline and flex‐fuel vehicle engine fuel standards.^[22]^ A rather straightforward extension of that method would be the design of dual‐use fuels, that is, renewable fuels that in their pure form aim to exploit the efficiency and emissions potential of dedicated engines and in a blended state with a fixed amount of gasoline aim for compatibility with today's conventional engines. In the longer term, the integration of reduced‐order engine models into the optimization problem may allow consideration of fuel/engine interactions and thus contribute to further improvements in engine efficiency.^[37]^ Another possible point of improvement is the harmonization of the global warming impact estimation in process network flux analysis (PNFA) and LCA. Furthermore, to expand on the green toxicology approach, possible blend constituents should not only be pre‐screened for human toxicity but also for ecotoxicity. Going beyond high‐efficiency spark‐ignition engines, model‐based fuel design should be applied also to other advanced combustion systems, including pre‐chamber combustion and dual‐fuel engines.

## Conflict of interest

The authors declare no conflict of interest.

## Supporting information

As a service to our authors and readers, this journal provides supporting information supplied by the authors. Such materials are peer reviewed and may be re‐organized for online delivery, but are not copy‐edited or typeset. Technical support issues arising from supporting information (other than missing files) should be addressed to the authors.

Supporting Information
