# Supplementary material for: Designed to Be Green, Economic, and Efficient: A Ketone‐Ester‐Alcohol‐Alkane Blend for Future Spark‐Ignition Engines
Source: ChemSusChem. 2021 Nov 12;14(23):5254–64. doi: 10.1002/cssc.202101704 (PMC9299169; doi:10.1002/cssc.202101704)
Supplement: Supplementary file 1 — Supporting Information [file CSSC-14-5254-s001.pdf]

ChemSusChem

Supporting Information

Designed to Be Green, Economic, and Efficient: A Ketone-Ester-Alcohol-Alkane Blend for Future Spark-Ignition Engines

Philipp Ackermann, Karsten E. Braun, Patrick Burkardt, Sebastian Heger, Andrea König, Philipp Morsch, Bastian Lehrheuer, Maximilian Surger, Simon Völker, Lars Mathias Blank, Miaomiao Du, Karl Alexander Heufer, Martina Roß-Nickoll, Jörn Viell, Niklas von der Aßen, Alexander Mitsos, Stefan Pischinger, and Manuel Dahmen*This publication is part of a collection of invited contributions focusing on "The Fuel Science Center-Adaptive Conversion Systems for Renewable Energy and Carbon Sources". Please visit "" to view all contributions.© 2021 The Authors. ChemSusChem published by Wiley-VCH GmbH. This is an open access article under the terms of the Creative Commons Attribution License, which permits use, distribution and reproduction in any medium, provided the original work is properly cited.

Evaluation of Production Process Performance

The production performance of KEAA, ethanol, 2-butanone, and EBCC was evaluated with PNFA.[1,2,3] In PNFA, the optimal configuration of production pathways is identified for a given reaction network, pre-defined economic and ecological parameters, and a fixed product composition, i.e., in contrast to the integrated fuel design method described in Section “Design of optimal renewable fuels: The KEAA blend and its production performance” of the manuscript, the product composition is not a degree of freedom. We used the same production pathway network and the same economic and ecological parameters that were published in the Supporting Information by König et al.[3] The optimization problem was implemented and solved using the software, solver, and solver settings as described in Section “Design of optimal renewable fuels: The KEAA blend and its production performance” of the manuscript and, more detailed, by König et al.[3]

Rapid Compression Machine

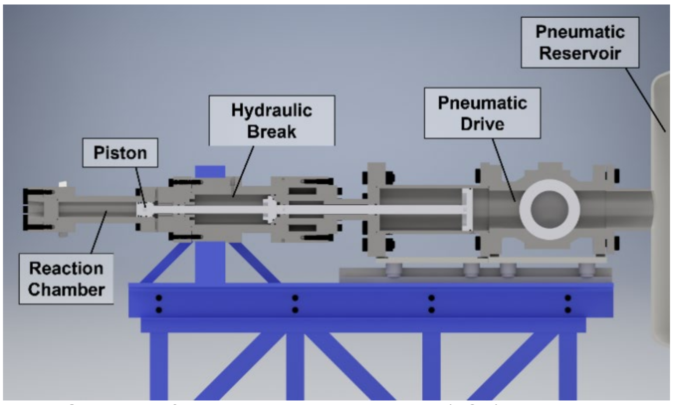

Figure S1: Layout of the rapid compression machine (RCM)

Ignition delay times longer than 2 ms and up to 200 ms have been measured in a rapid compression machine that was presented in detail by Lee et al. and Ramalingam et al.[4, 5] Briefly described, the RCM features a horizontal and cylindrical reaction chamber with an inner diameter of 50 mm as can be seen in Figure S1. A piston assembly with a total stroke of 250 mm is used to rapidly compress the gas inside the reaction chamber and thus leads to an auto-ignitable state. A manifold system is attached to the reaction chamber, which is used to create and store the gaseous mixture of interest. Both the manifold system and the reaction chamber were heated to 75 °C during the experimental series to avoid local condensation issues. A Kistler pressure transducer (6125C-U20) was used to determine the end-of-compression pressure as well as to detect the ignition event that was defined as the point in time with the maximum increase of pressure with respect to the time. The time interval between these events was defined as the ignition delay time (cf. Figure S2). A variable end wall design was used to change the compression ratio and thus end-of-compression temperature, which was calculated under the assumption of an isentropic compression inside the core gas of the reactor.[6] The end-of-compression pressure was varied by adjusting the initial pressure in the reaction chamber accordingly. Nevertheless, different bath gas compositions were needed to cover the temperature range of interest for the different pressures and fuel-air equivalence ratios investigated in this study. Although the dilution was always air-like ($x_{Diluent}/x_{O2}$ = 3.762), different ratios of N_2 and Ar were used for the diluent gas to adjust the mixture's heat capacity and thus the isentropic compression behavior.

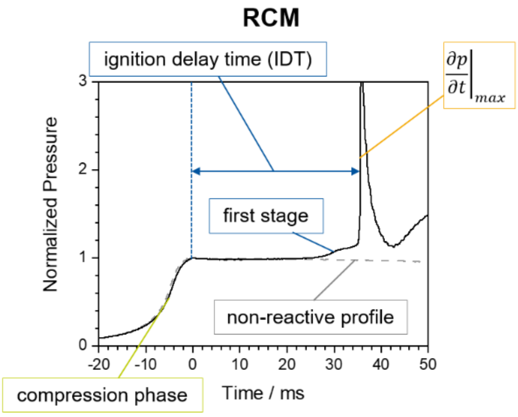

Figure S2: Generic pressure trace and definition of the ignition delay time in the rapid compression machine (RCM).

Single-Cylinder Research Engine

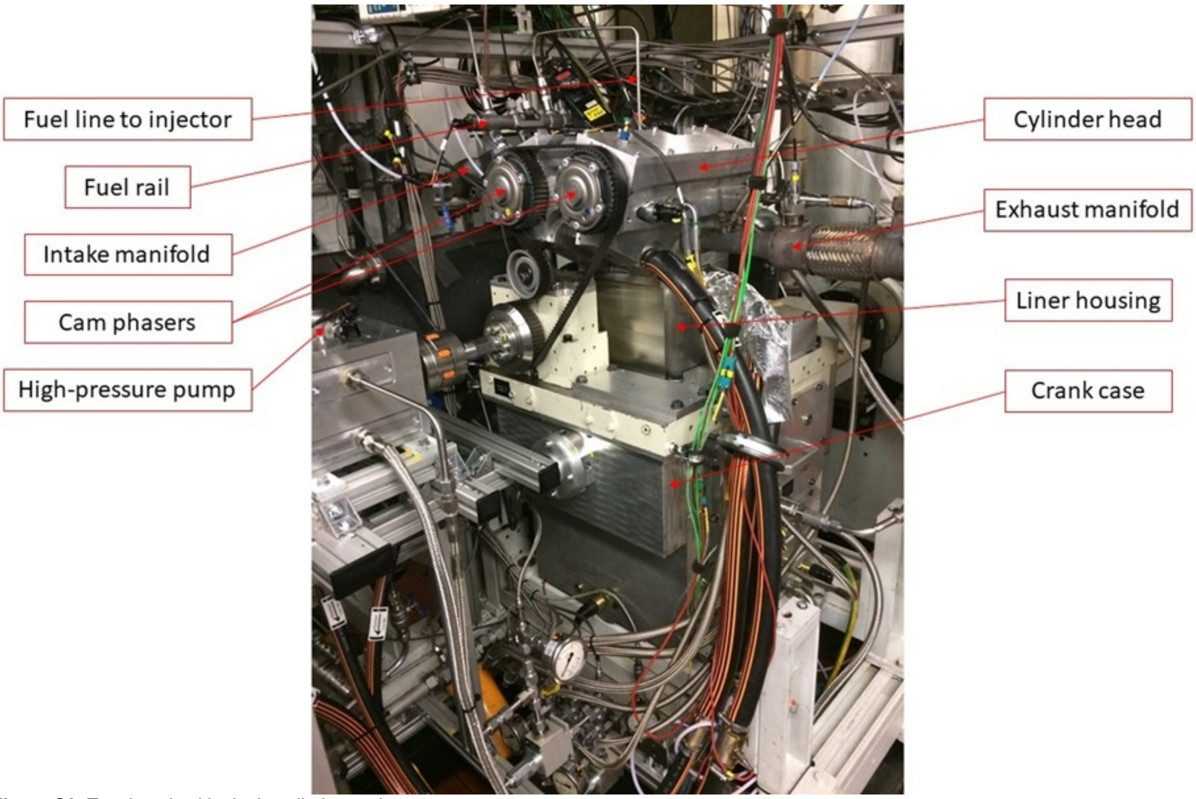

Figure S3: Test bench with single-cylinder engine.

All fuels were thermodynamically investigated on a direct-injection spark-ignition single-cylinder research engine (SCE) shown in Figure S3 to evaluate their combustion performance. In particular, the SCE is equipped with a laterally mounted direct injector and a centrally mounted spark plug and has a compression ratio of CR = 16.4 to exploit the potential benefits of the fuels regarding their expected knock-resistance. The detailed engine hardware specifications are tabulated in Table S1.

Table S1. Single-cylinder research engine hardware specifications.

	Unit	Value
Displacement	cm^3	500
Bore	mm	75
Stroke	mm	113.2
Connecting rod length	mm	220
Valves per cylinder	1	4
Intake valve event length[a]	° CA	178
Exhaust valve event length[a]	° CA	178
Max. intake valve lift	mm	8.8
Max. exhaust valve lift	mm	8.4
Compression ratio	1	16.4
Injector position	-	lateral
Spark plug position	-	central

[a] referred to 1 mm valve lift.

A variation of the indicated mean effective pressure (IMEP) at an engine speed of n = 2000 1 min^{-1} and a relative air-fuel ratio of λ = 1.0 was performed. The exhaust gas λ was derived according to the formula of Spindt with the extension for oxygenated fuels of Bresenham et al. considering the fuel specifications and the measured exhaust gas composition. [7,8] The exhaust gas composition was analyzed from a partial mass flow of the exhaust gas, which was sampled before an exhaust gas backpressure valve. The analyzed exhaust gas components are listed in Table S2.

Table S2. Measurement principles used for the exhaust gas composition measurements.

Pollutant	Measurement principle
Unburned hydrocarbons (HC)	Flame ionization detector (Rosemount NGA 2000)
Oxygen (O_2)	Paramagnetic oxygen analyzer (Rosemount NGA 2000)
Carbon monoxide (CO)	Infrared gas analyzer (Rosemount NGA 2000)
Carbon dioxide (CO_2)	Infrared gas analyzer (Rosemount NGA 2000)
Nitrogen oxides (NO_X)	Chemiluminescence analyzer (Eco Physics 700 EL ht)

The load control to achieve the desired IMEP was realized by a throttle valve at low loads and an external boosting device at high loads. In particular, the throttle valve was used up to an intake pressure of p_{in} = 1 bar. At p_{in} > 1 bar, the throttle valve was always fully opened and the required boost pressure was delivered by the external boosting device.
The exhaust gas backpressure was adjusted depending on the intake pressure via an additional flap in the exhaust pipe. When the intake pressure was p_{in} ≤ 1 bar, the backpressure flap was fully opened and consequently the exhaust pressure was p_{ex} = 1 bar. At p_{in} > 1 bar, the exhaust pressure was equal to the intake pressure (p_{ex} = p_{in}). The intake temperature was set to T_{in} = 25 °C.
The SCE is equipped with two pressure transducers in the pent roof to measure the in-cylinder pressure. Furthermore, two pressure transducers are mounted in the intake and exhaust manifolds, one in each manifold (see Table S3 for details). For each measuring point, 200 consecutive cycles were measured with a crank angle resolution of 0.1° CA. The combustion phasing, the burn delay, and the burn duration were calculated from the average of the two pressure transducers in the combustion chamber.

Table S3. Pressure transducers used for the in-cylinder pressure and transient intake and exhaust pressure measurements.

Combustion chamber	Two Kistler 6044A
Intake manifold	Kistler 4045A
Exhaust manifold	Kistler 4045A

Both the injection pressure (p_{rail}) and the start of fuel injection (SOI) were kept constant at p_{rail} = 200 bar and SOI = 300° CA bTDC, respectively. Also, the temperatures of both oil and coolant were constant at 90 °C at the engine outlet.

Ecotoxicity

***Daphnia magna* husbandry**: Single individuals of *D. magna* STRAUS (clone 5) were kept in in approx. 80 mL of Elendt M4 medium at 20 ± 1 °C and a 16:8 h light:dark cycle.[9] Feeding occurred three times a week with *Desmodesmus subspicatus* and once a week with commercial dry yeast. The medium was changed weekly.

Testing procedure: The acute immobilization tests was performed according to the OECD guideline for testing of chemicals 202, using *D. magna*.[9] Due to the inherent properties of the mixture, some modifications were necessary in accordance with the guideline on aqueous-phase aquatic toxicity testing of difficult test chemicals.[10] Regarding the potential adherence of constituents to plastic surfaces glassware was used. To reduce evaporative losses of constituents with a high vapor pressure, the test was performed in sealed 20 ml glass vials with PTFL lined metal caps. The exposure solution was prepared using gas-tight Hamilton syringes while reducing air contact prior to mixing by rotation by injecting the blend into a tilted volumetric flask containing M4 medium. *D. magna* neonates (age <24 h) were, additionally to a negative control (pure medium), exposed to five or six concentrations (KEAA: 50, 125, 200, 275, 350, 425 mg l^{-1}; EBCC: 48.5, 56.5 64.5, 72.5, 80.5 mg l^{-1}) with four vessels containing five neonates for each concentration. The test was repeated in three (EBBC) and four (KEAA) replicates. Additionally, a single replicate set of four vials at 500 mg l^{-1} of KEAA was included. The selection of test concentrations was based on prior range finding experiments and model predictions. The experimental setup was kept in identical conditions as the husbandry. After 24 h and 48 h immobility was documented. Neonates unable to swim within 15 s after gentle agitation were considered immobile. The validity criterion of 10 % immobility or less in the negative control was met for each experiment.

Data analysis: Data analysis was performed using the software ToxRat Professional Version 3.3. The dose-response curve was calculated by probit analysis using linear maximum likelihood regression.

Concentration-response curves: The concentration-response curves of KEAA and EBCC (see Figure S4) show their immobilizing effect on Daphnia magna in dependence of the tested concentrations. The fitted curve enabled the determination of ECx values, with EC50 being the concentration at which 50 % effect, here immobility, occur. Concentrations to the left of each curve (below the lowest observable effect concentration) have not been observed to induce adverse effects. While this single-species assay cannot be used to safely deduce harmless environmental concentrations, it serves as an indicator to the orders of magnitude in which adverse effects are to be expected and as a comparative measure for other fuels. The comparatively high EC50 of KEAA and therefore low toxic potency is, especially in comparison to conventional fuels, promising.

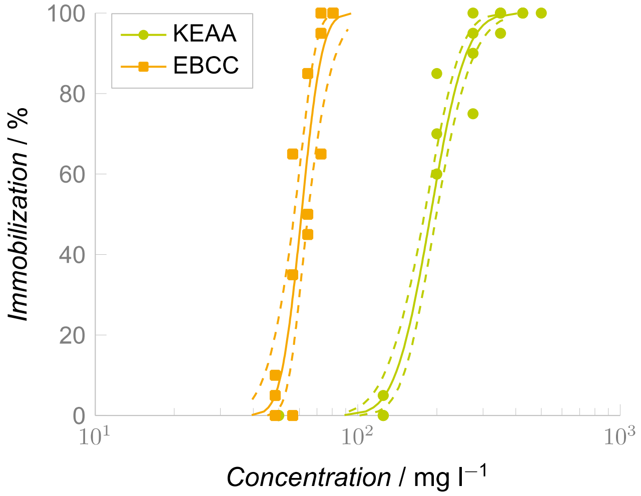

Figure S4: Percentage of immobilization at various concentrations of KEAA and EBCC in the acute daphnia immobilization assay. Dots indicate measured immobilization at the given concentration, lines indicate the respective concentration response curve used to determine EC values, dotted lines indicate the lower/higher 95 % confidence level.

Microbial Storage Stability

Microbial strains and growth conditions for assessment of microbial storability: To mimic a fuel tank, 50 ml of free water phase, consisting of 0.1 % NaCl, were overlaid with about 250 ml gasoline or biodiesel (RME) within a 500 ml shot bottle, resulting in a 300 ml headspace (storage culture). Valves were used between the opening of the shot bottle and the CO_2 sensor to enable the sensors to be relocated between culture bottles. A volume of 1400 ml water phase was inoculated with a mixture of 20 microbes representative of contaminated fuel storage as defined by Leuchtle et al. [11] including an amount per strain corresponding to 32 mg CDW (cell dry weight) (a total of 640 mg for all microbes) (Figure S5). The precultures were made at 30°C and 200 rpm in LB (lysogeny broth) medium, YEP (yeast extract peptone) medium, potato extract glucose bouillon (PEGB, 26,5 g L^{-1}), and ME (malt extract) medium for bacteria, yeasts, *Rhodotorula mucilaginosa*, and molds. The precultures were washed with 0,1 % NaCl before use. Table S4 shows a summary of all strains. The shot bottles were not shaken and were kept in the dark. The storage cultures were incubated at room temperature.

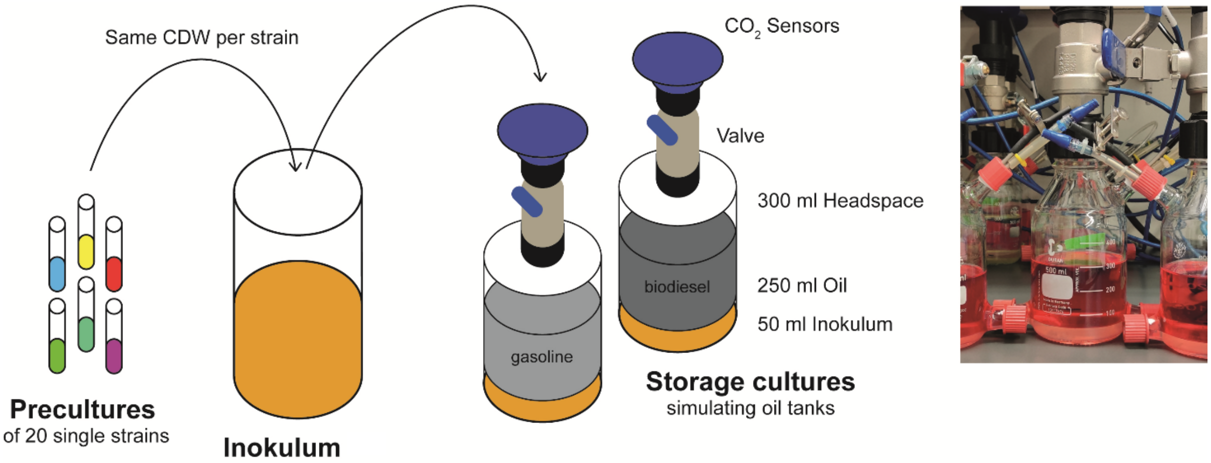

Figure S5: The setup of the storage cultures, used for the assessment of microbial storability, is shown. The free water phase of all storage cultures, including different oil phases, were prepared as one masterbatch of 0,1% NaCl, which was inoculated with equal amounts of 20 microbes (normalized by CDW), which are representative for fuel contamination (defined by Leuchtle et al.).[11] An aliquot of 50 ml is transferred into each storage culture and overlayed with 250 ml of gasoline or biodiesel, resulting in a headspace of 300 ml. The culture bottle is sealed airtight with valve or valve plus CO2 sensor.

CO_2 measurement as indication of microbial activity: For the measurement of CO_2 development, we used the BCP-CO_2 system (BlueSens Gas Sensor GmbH). The CO_2 sensor inhabits a source of infra-red light, which is weakened by the analyte gas and reflected into the detector unit of the sensor. The sensor was attached airtight to the opening of a culture vessel, which was in this study a 500 ml shot bottle. Measurements were done without air-exchange, so that a measurement of microbial activity beyond two to three weeks is not possible with the oxygen available in the culture bottle.
HPLC measurement: To show which alcohol concentrations the microbes are confronted with during the storage culture, the free water phase of control samples was harvested after one week and analyzed by HPLC without further treatment. The control samples consisted of 2 ml 0.1 % NaCl without microbes under 10 ml of fuel in 12 ml glass vials. An UltiMate 3000 series instrument from Dionex-Themo Scientifc was used for HPLC analysis. A Metab-AAC HPLC column of the BF series from ISERA (300 x 8 mm), intended for the analysis of sugars, organic acids and alcohols, was used. 5 mM H_2SO_4 was applied as running buffer at a flow rate of 0.6 ml/min. The column oven was kept at 75°C. The alcohols were detected via the IR detector unit Refracto Max 521 (running at 35°C with a data collection rate of 10 Hz and an integrator range of 500 µRIU V^{-1}). Quantification was based on peak areas and occurred via an external standard curve of the corresponding alcohol.

Table S4. Strains used in this study and for the defined inoculum of the fuel tank simulation.[11]

Strain	Database number	Source
Acinetobacter beijernikii	DSM 22901	DSMZ
Acinetobacter venetianus	DSM 23050	DSMZ
Burkholderia cepacia	DSM 7288	DSMZ
Micrococcus luteus	-	Leuchtle et al. (2018)[11]
Micrococcus yunnanensis	DSM 21948	DSMZ
Pseudomonas fluorescens	-	Leuchtle et al. (2018)[11]
Pseudomonas poae	-	Leuchtle et al. (2018)[11]
Candida cylindracea	DSM 2031	DSMZ
Debaryomyces hansenii	DSM 70244	DSMZ
Debaryomyces polymorphus	DSM 70816	DSMZ
Pichia membranifaciens	DSM 21959	DSMZ
Raffaelea sp.	-	Leuchtle et al. (2018)[11]
Rhodotorula mucilaginosa	DSM 18184	DSMZ
Ustilago maydis	-	Leuchtle et al. (2018)[11]
Yarrowia deformans	CBS 2071	DSMZ
Yarrowia lipolytica	-	Leuchtle et al. (2018)[11]
Paecilomyces lilacinus	DSM 846	DSMZ
Penicillium chrysogenum	DSM 21171	DSMZ
Penicillium citrinum	-	Leuchtle et al. (2018)[11]
Micrococcus luteus	-	Leuchtle et al. (2018)[11]

Life Cycle Assessment

With life cycle assessment (LCA), environmental impacts of product systems can be analyzed along the entire life cycle. LCA considers every material and energy flow that is exchanged with the environment and is standardized in ISO 14040/14044.[12,13]

The goal of the presented well-to-wheel LCA was to compare KEAA, 2-butanone, EBCC, and ethanol to fossil gasoline in terms of the carbon footprint. To compare all fuels consistently, we defined the "provision of 1 MJ of enthalpy of combustion" as the so-called functional unit. Thus, we assumed for simplicity that all fuels could be used with equal efficiencies in a spark-ignition engine in the use-phase of the fuels. For the end-of-life, we considered the combustion of wastes and by-products lignin and hemicellulose in addition to fuel combustion. We evaluated as environmental impact category the carbon footprint in g CO_2-eq. and used the Environmental Footprint 2.0 methodology recommended by the Joint Research Center of the European Commission.[14]

We differentiate between a best-case and a worst-case scenario in terms of the carbon footprint. The scenario-specific technologies lead to the lowest and the highest carbon footprint, respectively. The best-case scenario uses wind power for electricity supply and the electricity-based technologies heat pumps and electrode boilers for heat supply.[15] The heat pump was modeled with a coefficient of performance (COP) of 3.28 that was estimated from heat pumps built since 2006, which have evaporator temperatures in the range of 9 to 15 °C and condenser temperatures of about 90 °C.[16] The electrode boiler was modeled with a Power-to-Heat efficiency of 95 %.[17] Instead in the worst-case scenario, electricity is supplied by today's power grid mix and heat is supplied by steam in the chemical industry and natural gas boilers.[15]

The mass of required cooling water was calculated from the cooling demand, the specific heat capacity of water (4.18 kJ kg^{-1} K^{-1}), and a temperature difference for the cooling water of 10 K. We modeled the electricity consumption to provide refrigeration as the average of a pulse tube cryocooler and a Stirling cryocooler.[18] The COPs of both cryocoolers are calculated as functions of the refrigeration temperature and for an ambient temperature of 300 K. The underlying functions are based on data from a cryocooler survey. For biomass, we assumed beech wood from hardwood forestry.[15] For fossil gasoline, we assumed a lower heating value of 41.3 MJ per kg gasoline and a carbon content of 0.83 kg carbon per kg gasoline. We considered the E10 gasoline mix at German filling stations as LCA data set.[19] Additional process data sets, i.e., for wastewater treatment and process water supply, were taken from the ecoinvent LCA database.[15] For more details on the used LCA data sets, see Table S5.

Table S5. LCA data sets used in this study.

Flow	Data set	Region	Reference
Biomass	Hardwood forestry, beech, sustainable forest management	DE	Ecoinvent 3.7.1 cut-off [15]
Process water	Market for water, decarbonized	DE	Ecoinvent 3.7.1 cut-off [15]
Wastewater	Treatment of wastewater, average, capacity 1E9 l $year^{-1}$	Europe	Ecoinvent 3.7.1 cut-off [15]
Gasoline	Gasoline mix (E10) at filling station		Gabi [19]
Electricity	Market for electricity, medium voltage	DE	Ecoinvent 3.7.1 cut-off [15]
	Electricity production, wind, 1-3 MW turbine, onshore	DE	Ecoinvent 3.7.1 cut-off [15]
Heat < 100°C	Steam production, as energy carrier, in chemical industry	Europe	Ecoinvent 3.7.1 cut-off [15]
	Heat pump (COP = 3.28)		David et al, 2017 [16]
100°C < Heat < 250°C	Steam production, as energy carrier, in chemical industry	Europe	Ecoinvent 3.7.1 cut-off [15]
	Electrode boiler (η = 95 %)		Müller et al, 2020 [17]
Heat > 250°C	Heat production, natural gas, at boiler modulating >100 kW	Europe	Ecoinvent 3.7.1 cut-off [15]
	Electrode boiler (η = 95 %)		Müller et al, 2020 [17]

References

[1] K. Ulonska, M. Skiborowski, A. Mitsos, J. Viell, *AIChE J.* **2016**, *62*, 3096-3108.

[2] A. König, K. Ulonska, A. Mitsos, J. Viell, *Energ. Fuel.* **2019**, *33*, 1659-1672.

[3] A. König, M. Siska, A. M. Schweidtmann, J. G. Rittig, J. Viell, A. Mitsos, M. Dahmen, *Chem. Eng. Sci.* **2021**, *237*, 116562.

[4] C. Lee, S. Vranckx, K. A. Heufer, S. V. Khomik, Y. Uygun, H. Olivier, R. X. Fernandez, *Z. Phys. Chem.* **2012**, *226*, 1-28.

[5] A. Ramalingam, K. Zhang, A. Dhongde, L. Virnich, H. Sankhla, H. Curran, A. Heufer, *Fuel* **2017**, *206*, 325-333.

[6] C.-J. Sung, H. J. Curran, *Prog. Energ. Combust.* **2014**, *44*, 1-18.

[7] R. S. Spindt, *SAE Tech. Pap.* **1965**, 788-793.

[8] D. Bresenham, J. Reisel, K. Neusen, *SAE Tech. Pap.* **1998**, 2154-2171.

[9] OECD, Test No. 202: Daphnia sp. Acute Immobilisation Test, OECD Guidelines for the Testing of Chemicals, Section 2, **2004**.

[10] OECD, Guidance Document on Aquatic Toxicity Testing of Difficult Substances and Mixtures, OECD Series on Testing and Assessment, **2019**.

[11] B. Leuchtle, L. Epping, W. Xie, S. J. Eiden, W. Koch, D. Diarra, K. Lucka, M. Zimmermann, L. M. Blank, *Int. Biodeter. Biodegr.* **2018**, *132*, 84-93.

[12] International Organization for Standardization, ISO 14040: Environmental Management: Life Cycle Assessment: Principles and Framework, **2006**. Available from https://www.iso.org/standard/37456.html (accessed May 2021)

[13] International Organization for Standardization, ISO 14044: Environmental Management: Life Cycle Assessment: Requirements and Guidelines, **2006**. Available from https://www.iso.org/standard/38498.html (accessed May 2021)

[14] European Commission, *PEFCR Guidance document*: "Guidance for the development of Product Environmental Footprint Category Rules (PEFCRs), version 6.3, December 2017", **2017**. Available from https://ec.europa.eu/environment/eussd/smgp/pdf/PEFCR_guidance_v6.3.pdf (accessed May 2021)

[15] G. Wernet, C. Bauer, B. Steubing, J. Reinhard, E. Moreno-Ruiz, B. Weidema, *Int. J. Life Cycle Assess.* **2016**, *21*, 1218-1230.

[16] A. David, B. V. Mathiesen, H. Averfalk, S. Werner, H. Lund, *Energies* **2017**, *10*, 578.

[17] L. J. Müller, A. Kätelhön, S. Bringezu, S. McCoy, S. Suh, R. Edwards, V. Sick, S. Kaiser, R. Cuéllar-Franca, A. El Khamlichi, J. H. Lee, N. von der Assen, A. Bardow, *Energ. Environ. Sci.* **2020**, *13*, 2979-2992.

[18] D. R. Ladner, *Proc. 16th Int. Cryocooler Conf.* (Atlanta, GA) **2008**, 633-644.

[19] Sphera Solutions, Inc., "Gabi: Software-System and Database for Life Cycle Engineering, Database version 2021.1", (Accessed May 2021).
